# Embryonic paratesticular rhabdomyosarcoma: a case report

**DOI:** 10.1186/1752-1947-7-93

**Published:** 2013-04-05

**Authors:** Ahmed Amine Bouchikhi, Soufiane Mellas, Mohammed Fadl Tazi, Karim Lahlaidi, Youssef Kharbach, Khadija Benhayoune, Rajae Kanab, Jalal Eddine Elammari, Abdelhak Khallouk, Mohammed Jamal El Fassi, My Hassan Farih

**Affiliations:** 1 Service d’Urologie, Centre Hospitalier Universitaire de Fès, Fès, Maroc

## Abstract

**Introduction:**

An embryonic paratesticular rhabdomyosarcoma is a very rare mesenchymal tumor. It is an intrascrotal tumor that is localized in paratesticular structures such as the epididymis or spermatic cord. Rhabdomyosarcoma is most often observed in children and adolescents, presenting as a painless scrotal mass.

**Case presentation:**

Our patient was an 18-year-old Moroccan man who presented with a painless left scrotal mass that had evolved over four months. An inguinal orchiectomy was performed. A histological examination of the excised tissue revealed an embryonic rhabdomyosarcoma.

Our patient had three sessions of chemotherapy with vincristine, actinomycin C and cyclophosphamide. Each chemotherapy session was conducted over five days, with a cycle of 21 days. Our patient was assessed two months after the last chemotherapy session and demonstrated good clinical improvement.

**Conclusion:**

Paratesticular rhabdomyosarcoma is a rare aggressive tumor manifesting in children and very young adults. Localized forms have a good prognosis whereas metastatic tumors show very poor results. A well-defined treatment based on surgery and chemotherapy yields good results.

## Introduction

An embryonic paratesticular rhabdomyosarcoma is a very rare mesenchymal tumor. It is an intrascrotal tumor that is localized in paratesticular structures such as the epididymis or spermatic cord. Rhabdomyosarcoma is most often observed in children and adolescents where it is usually presents as a painless scrotal mass [1].

We report the case of an 18-year-old man who presented with a large mass that had developed over four months. An investigation into tumoral markers was negative. The surgical intervention consisted of left orchiectomy. A histological examination of the surgical specimen confirmed embryonic paratesticular rhabdomyosarcoma. Adjuvant chemotherapy was initiated.

## Case presentation

Our patient was an 18-year-old Moroccan man who presented with a painless left scrotal mass that had evolved over four months. A clinical examination revealed a hard testicular mass in his left side with a diameter of 10cm; the mass was renitent, static and suspicious.

Clinical examinations of his lymph nodes and abdomen were normal. An ultrasound revealed an expansive process characterized by intrascrotal heterogeneous tissue density. A computed tomography (CT) scan of his thorax, abdomen and pelvis showed a left heterogeneous testicular tumoral process with high adherence measuring 7cm to 8cm. However, neither pelvic nor lombo-aortic lymphadenopathy was revealed. The thoraco-abdomino-pelvic CT scan did not show any metastases.

Our patient’s levels of tumoral markers alpha-fetoprotein, beta-human chorionic gonadotropin and angiotensin-converting enzyme were normal. An inguinal orchiectomy was performed. A histological examination of the surgical specimen demonstrated tumoral proliferation with unorganized architecture in sheets of pleomorphic clear tumoral cytoplasmic cells and eosinophils with atypical nuclei; eccentric aspects with strong nucleoli and multi-nucleoli with rhabdomyoblastic aspects were observed. This confirmed the diagnosis of an embryonic rhabdomyosarcoma.

**Figure 1:**
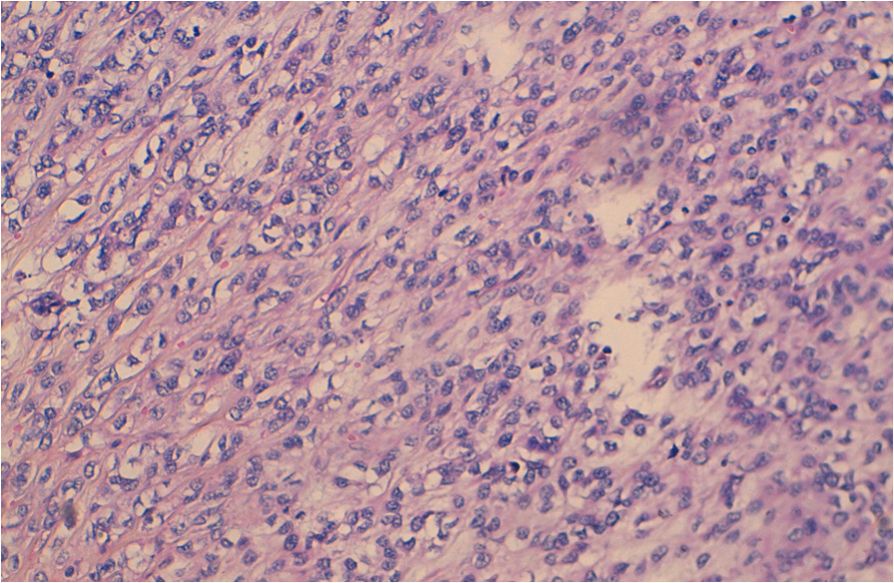
**Histological aspects of embryonic paratesticular rhabdomyosarcoma ×20 magnification.** (Hematoxylin and eosin stain).

**Figure 2:**
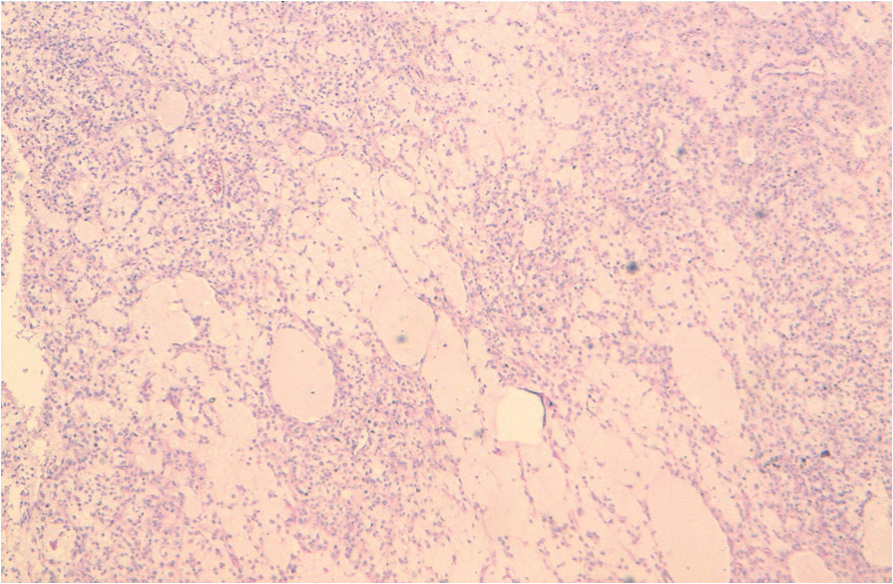
**Histological aspects of embryonic paratesticular rhabdomyosarcoma, ×50 magnification.** (Hematoxylin and eosin stain).

Immunohistochemistry demonstrated focal myogenic expression in less than half of the tumoral cells (Figures 1 and 2).

Three chemotherapy sessions of vincristine 1.5 mg/m^2^, actinomycin C 1.5mg/m^2^ and cyclophosphamide 500 mg/m^2^ were performed. Each chemotherapy session was conducted over five days, with a cycle of 21 days. Our patient was assessed two months after his last chemotherapy session and demonstrated good clinical improvement.

## Discussion

Rhabdomyosarcoma usually occur in the extremities. A paratesticular localization is rare; the tumor develops from mesenchymal tissues of the spermatic cord, and epididymis.

There are two frequency peaks found for the development of rhabdomyosarcoma, the first at the age of 4 years and the second at the age of 18 years, as in our case. There is no predilection for race [2].

The consensus is that this tumor derives from mesenchymal elements of the testis envelope, epididymis and spermatic cord. The tumor manifests as a hard painless inguinoscrotal swelling, the size and duration of development are varied and it rarely invades the scrotal skin. The mass might evolve within the external inguinal ring away from the scrotal contents [2,3]. A clinical examination should be complemented by an examination of the lymph nodes and a general examination to investigate metastases. Differential diagnoses include testicular torsion, orchiepididymitis, scrotal abscess and, rarely, testicular tuberculosis.

A testicular ultrasound is routinely performed for a scrotal mass. This imaging modality shows a mass with heterogeneous echogenicity and inguinoscrotal extension in 80% of cases [4]. This allows the nature of the intrascrotal tissue mass to be determined and specifies the exact topography. Ultrasound is used to establish the differential diagnosis and eliminate diagnoses of simple cyst and varicocele.

A thoraco-abdomino-pelvic CT scan allows for any deep invasion of the lymph nodes to be investigated, especially lombo-aortic and pelvic metastases as well as possible metastases to the liver or lung. Most authors prefer the use of thoraco-abdomino-pelvic CT compared to ultrasound for lymphomas.

Magnetic resonance imaging is an efficient imaging modality when using surface coils. The tumor appears homogeneous in T1-weighted images and heterogeneous in T2-weighted images with signal intensity similar to that in a normal testis. The low signal intensity of the tunica albuginea in T2-wieghted images allows the visualization of a clear separation of the mass from the testis [1,5,6].

In rhabdomyosarcoma, tumoral markers including alpha-fetoprotein, beta-human chorionic gonadotropin and carcinoembryonic antigen are usually normal. This was the case with our patient.

A malignant tumor might be suspected in masses sitting in the distal cord with a hard and irregular form adhering to surrounding structures; a rapid increase in tumor volume might be noted. But the diagnosis is mainly made using histology. The macroscopic features include a lobed tumor surrounded by whitish pseudocapsules, hemorrhagic array are sometimes revealed [7].

There are four histological types of rhabdomyosarcoma: pleomorphic, alveolar, botryoidal, and embryonal, such as found in our patient. The characteristic cell is rhabdomyoblast, which is not necessary for the diagnosis. Whenever rhabdomyoblasts are not present, immunohistochemical investigations are conducted using a panel of antibodies including myosin and desmin [6,8,9].

Rhabdomyosarcoma may be included in the differential diagnosis for other paratesticular sarcomas such leiomyosarcoma, liposarcoma and fibrosarcoma. However, these pathologies occur more often in adults. Imaging cannot discriminate between these tumors. The final diagnosis can be established by histological study after surgical excision of the tumoral mass [6,7].

Radical orchidectomy by the inguinal route with first cord ligation remains the essential act for histological diagnosis and constitutes the first step of treatment regardless of the stage of the disease. Hemiscrotectomy associating inguinal treatment is indicated first in scrotal cases whenever local invasion or presence of lymph are clinically evidenced [6,7]. An inguinal lymphadenectomy should not be performed without first obtaining imaging, including a CT scan or lymphography.

Chemotherapy should be routinely administered since rhabdomyosarcoma is chemosensitive. This therapeutic approach consists of administrating actinomycin D, vincristine and cyclophosphamide [5-7,10]. Radiotherapy is a complementary treatment of chemotherapy and surgery to eliminate residual foci and retroperitoneal lymph nodes.

Our patient benefited from an inguinal orchiectomy. Three sessions of chemotherapy were performed, resulting in no signs of metastasis or residual foci.

## Conclusion

Paratesticular rhabdomyosarcoma is a rare aggressive tumor manifesting in children and very young adults. Localized forms have a good prognosis whereas metastatic tumors show very poor results. A well-defined treatment based on surgery and chemotherapy yields good results. Radiotherapy is indicated in cases of residual foci and retroperitoneal lymphnodes. Strict follow-up has to be instituted for all patients.

## Consent

Written informed consent was obtained from the patient for publication of this case report and any accompanying images. A copy of the written consent is available for review by the Editor-in-Chief of this journal.

## Competing interests

The authors declare that they have no competing interests.

## Authors’ contributions

AAB was the principal author and major contributor in writing the manuscript. SM, MFT, RK, KL, KB, JE, AK and YK analyzed and interpreted the patient data and reviewed the literature. MJE and MHF read and corrected the manuscript. All authors read and approved the final manuscript.
